# Surface Modification of Polycaprolactone Scaffold With Improved Biocompatibility and Controlled Growth Factor Release for Enhanced Stem Cell Differentiation

**DOI:** 10.3389/fbioe.2021.802311

**Published:** 2022-01-07

**Authors:** Xiaoyan Qin, Yixin Wu, Shuang Liu, Lei Yang, Hongxia Yuan, Susu Cai, Julia Flesch, Zehao Li, Yujing Tang, Xiaomin Li, Yi Zhuang, Changjiang You, Chaoyong Liu, Changyuan Yu

**Affiliations:** ^1^ College of Life Sciences and Technology, Beijing University of Chemical Technology, Beijing, China; ^2^ Department of Spine Surgery, The First Affiliated Hospital, Shenzhen University, Shenzhen Second People’s Hospital, Shenzhen, China; ^3^ Department of Biology/Chemistry, University of Osnabrück, Osnabrück, Germany; ^4^ Center of Cellular Nanoanalytics (CellNanOs), Osnabrück, Germany; ^5^ SINOPEC, Beijing Research Institute of Chemical Industry, Beijing, China; ^6^ Science and Technology Department China Petrochemical Corporation, Beijing, China

**Keywords:** surface modification, polycaprolactone scaffold, controlled release, cell differentiation, growth factor

## Abstract

Polycaprolactone (PCL) has been widely used as a scaffold material for tissue engineering. Reliable applications of the PCL scaffolds require overcoming their native hydrophobicity and obtaining the sustained release of signaling factors to modulate cell growth and differentiation. Here, we report a surface modification strategy for electrospun PCL nanofibers using an azide-terminated amphiphilic graft polymer. With multiple alkylation and pegylation on the side chains of poly-L-lysine, stable coating of the graft polymer on the PCL nanofibers was achieved in one step. Using the azide-alkyne “click chemistry”, we functionalized the azide-pegylated PCL nanofibers with dibenzocyclooctyne-modified nanocapsules containing growth factor, which rendered the nanofiber scaffold with satisfied cell adhesion and growth property. Moreover, by specific immobilization of pH-responsive nanocapsules containing bone morphogenetic protein 2 (BMP-2), controlled release of active BMP-2 from the PCL nanofibers was achieved within 21 days. When bone mesenchyme stem cells were cultured on this nanofiber scaffold, enhanced ossification was observed in correlation with the time-dependent release of BMP-2. The established surface modification can be extended as a generic approach to hydrophobic nanomaterials for longtime sustainable release of multiplex signaling proteins for tissue engineering.

## Introduction

Ever since the definition of “tissue engineering” appears in the 1990s, polycaprolactone (PCL) nanofibers have been used as scaffold materials for tissue repair and regeneration due to their high stability, low cost, and biodegradability (Stafiej et al., 2017; Qi et al., 2021a; Qi et al., 2021b). By tuning the molecular weight of PCL, the physical and chemical properties, degradation efficiency, and mechanical strength are adjustable. PCL scaffold materials are therefore used to mimic the extracellular matrix (ECM), which provides mechanic support and cell signaling cues for cell proliferation and differentiation. Since the interactions between cells and scaffold materials occur at the scaffold surface, the ability of cells to undergo basic cellular processes (such as cell adhesion, migration, proliferation, and differentiation) at the scaffold interface is critical (Yang et al., 2019; Zhao et al., 2021). However, the high hydrophobicity of PCL seriously affects the adhesion and growth of cells on the material. Improving the biocompatibility of PCL is a demanding prerequisite for its application in tissue engineering.

Extensive efforts have been employed to modify the PCL scaffold surface with increased hydrophilicity, mainly including covalent chemical conjugation and non-covalent physical adsorption methods (Madhurakkat Perikamana et al., 2015; Zhang et al., 2018; Chuang et al., 2019). Chemical conjugation *via* the coupling of carboxylic acid group (COOH), amino group (NH_2_), alcohol group (OH), or alkynyl-azide “click chemistry” coupling, and sulfhydryl-maleimide coupling are often used to covalently attach hydrophilic functional groups to the surface of nanomaterial (Grafahrend et al., 2011; Lancuski et al., 2012; Lu et al., 2012; Han et al., 2014; Wade et al., 2015; Zhang et al., 2016). Oxygen plasma treatment, chemical etching, or γ-ray irradiation are also used to introduce biomolecules such as proteins, peptides, or growth factors on the surface (Chen et al., 2009; Boccafoschi et al., 2012; Levato et al., 2015). The covalent chemical conjugation methods ensure stable surface modification; however, they always require stringent reaction conditions and laborious multi-step procedures, and the risk of toxic by-products during the reaction. As for physisorption *via* van der Waals interactions, adhesion proteins such as fibrogenic protein, serum albumin, and other ECM proteins are commonly used to obtain the hydrophilic surfaces. However, these methods always suffer from long-term stability due to reverse dissociation of the bound proteins. Coating exogenous proteins also brings the risk of immune response and unwanted cell signal stimulation, leading to uncontrolled stem cell differentiation (Lin et al., 2006; Zhu et al., 2020; Geng et al., 2021b).

To establish reliable stem cell culture and differentiation, the scaffold not only needs to replicate the stiffness and biocompatibility of the ECM environments but also requires to comprise signaling ingredients in ECM and mimic their spatiotemporal regulations (Silva and Mooney, 2004; Jhala and Vasita, 2015). Growth factors are signaling substances that play essential roles for cell proliferation, which together with other stimulations jointly induce cell response to differentiate into the desired lineage (Chen et al., 2020; Liu et al., 2020). As tissue regeneration and repair often take a long time, controlled release of growth factors over weeks is crucial for tissue engineering of ossification (Geng et al., 2021a). Particularly, morphogens, such as the Wnt family members (Clevers and Nusse, 2012) and bone morphologic proteins (BMPs) (Mueller and Nickel, 2012), are tightly regulated within the stem cell niche for stem cell proliferation and differentiation. The BMPs form dimers (Ehrlich et al., 2011) in physiological conditions, which show a short-range effect by their abundant positive surface charges (Matsuda et al., 2016). Spatiotemporal control of BMP signal has been observed in different organs such as the intestine and hair follicle and play a determinant role in ossification (Rompolas et al., 2013; Carulli et al., 2014; Date and Sato, 2015). To protect the growth factors from environmental degradation and maintain their biological activity over a long time, hydrogel encapsulation has been used (Holland et al., 2005; Lee and Shin, 2007; Park et al., 2007; Morrell et al., 2008; Wan et al., 2008; Shachar et al., 2011; Yue et al., 2015). Controlling the release time of growth factors can be achieved by tuning the degree of cross-linking. However, a similar dilemma occurs as the physical encapsulation of signaling factors causes short-term release, while the chemical encapsulation may damage the activity by covalent bonding at the active sites of signaling proteins (Khan et al., 2014; Marchioli et al., 2016).

To obtain fully functional release, elegant designs of nanocarriers such as liposomes and micelles have been introduced to encapsulate growth factors and BMPs (Morrell et al., 2008; Ratanavaraporn et al., 2012). We previously developed a nanocapsule-based growth factor delivery platform by caging the proteins with a degradable polymer network. Different from liposomes and micelles that formed by non-covalent self-assembly of packing molecules, the polymer network can form a protecting layer around the internal growth factor and can be degraded to release the growth factor cargos over a long time. The rate of protein release can be controlled by adjusting the ratio of monomer to cross-linker (Tian et al., 2016; Xu et al., 2019).

**SCHEME 1:**
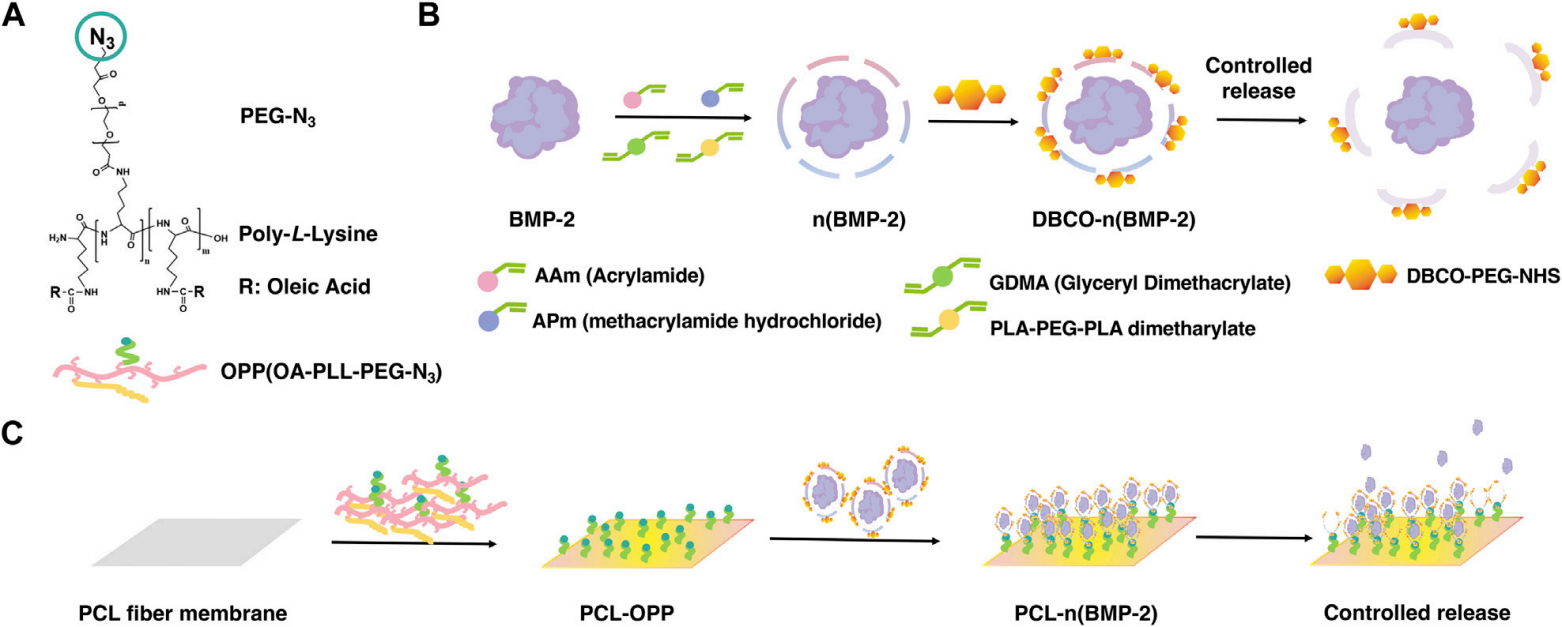
**(A)** Chemical structure of OA-PLL-PEG-N3 graft polymer. **(B)** Schematic illustration of the synthesis of BMP-2 nanocapsules and DBCO-modified BMP-2 nanocapsules. **(C)** Schematic illustration of surface modification of polycaprolactone scaffold with improved biocompatibility and controlled growth factor release. **(I)** Stable coating of hydrophobic polycaprolactone scaffold by OA-PLL-PEG-N_3_ graft polymer in one step. **(II)** Biofunctionalization of PLL-PEG-azide-coated scaffold with DBCO modified growth factor nanocapsules *via* click reaction. **(III)** Controlled release of growth factors from the nanocapsules-immobilized scaffold for inducing stem cell differentiation.

Here, we report a surface modification strategy for hydrophobic PCL scaffold using an azide-terminated amphiphilic polymer (Scheme 1A), which can be further functionalized with dibenzocyclooctyne (DBCO)-modified growth factor nanocapsules through the azide-alkyne “click chemistry”. The amphiphilic polymer was synthesized by grafting α-Carboxyl-ω-azido Poly (ethylene Glycol) (HOOC-PEG-N3, Mw 5,000 Da) and oleic acid to the side chains of poly-L-lysine (PLL, Mw 30–70 kDa) (Supplementary Figure S1A). The growth factor nanocapsules with controlled-release capability were synthesized using the methods as previously described where BMP-2 was used as a model growth factor. As shown in Scheme 1B, the synthesis was achieved through *in situ* polymerization of N-(3-aminopropyl) methacrylamide (APm), acrylamide (AAm), and glycerol dimethacrylate (GDMA, degradable cross-linker) and Poly (DL–lactide)–b–poly (ethylene glycol)–b–Poly (DL–lactide) diacrylate (PLA-PEG-PLA dimetharylate, degradable cross-linker) around the growth factors. Then, the BMP-2 nanocapsules [denoted as n(BMP-2)] were modified with DBCO-PEG-NHS for further conjugation with the azide group, which is denoted as DBCO-n(BMP-2). Controlled degradation of GDMA and PLA-PEG-PLA diacrylate under a neutral pH environment breaks the shells and enables the controlled release of BMP-2. With multivalent alkylation and PEGylation on the side chains, coating of the graft polymer on the PCL scaffold was achieved through the non-covalent interaction between the OA and PCL in one step (Step I), which is denoted as OA-PLL-PEG-N_3_ herein and after (Scheme 1C). The DBCO-n(BMP-2) was further conjugated with OA-PLL-PEG-N_3_ *via* click reaction (Step II). The pH-sensitive polymer network of the nanocapsules allows the controlled release of the growth factor and induces the differentiation of stem cells (Step III).

Through surface modification, the PCL is rendered biocompatible for cell adhesion and proliferation. Furthermore, the BMP-2 is protected by the pH-sensitive polymer network and immobilized on the modified PCL surface, allowing preserved activity on the scaffold and controlled release for differentiation of stem cells. As a proof of concept, BMP-2 was used in this work as a model growth factor to verify the effectiveness of our strategy for enhancing the ossification of bone mesenchyme stem cells (BMSCs). The results showed that quantitatively controlled release of active BMP-2 was achieved within 21 days. When BMSCs were cultured on the cell signaling nano fabric scaffold, enhanced ossification was observed correlating with the time-dependent release of BMP-2.

## Materials and Methods

### Reagents

Bone morphogenetic protein 2 (BMP-2) was obtained from Shanghai Ruibang Biomaterials Co., Ltd. (Shanghai China). PCL was purchased from Qingdao Nuokang Environmental Technology Co., Ltd. (Qingdao, China). PLL was obtained from Source leaf (Shanghai, China). Oleic acid was purchased from Sigma Aldrich (St. Louis, MO). HOOC-PEG-N_3_ (95%) was obtained from 3A Chemicals (Shanghai, China). DBCO-PEG-NHS N/A was purchased from Xi’an Ruixi Biotech (Xian, China). Deuterated methanol (99.8%) was purchased from Cambridge Isotope Laboratories (Ohio, United States). AAm was obtained from Aladdin, (Shanghai, China). N-(3-aminopropyl) methacrylamide hydrochloride (APm) was purchased from HEOWNS (Tianjin, China). N,N-methylenebisacrylamide (BIS) and ammonium persulfate (APS) were obtained from FuChen (Tianjin, China). N,N,N′,N′-tetramethylethylenediamine (TEMED) and Glyceryl Dimethacrylate (GDMA) were purchased from Sangon Biotech (Shanghai, China). Poly (DL–lactide)–b–poly (ethylene glycol)–b–Poly (DL–lactide) diacrylate (PLA–diacrylate, PLA:PEG:PLA = 2:24:2) was purchased from PolySciTech (West Lafayette, IN). Bovine serum albumin (BSA), BCA protein concentration determination kit, fluorescein isothiocyanate (FITC), dexamethasone, ascorbic acid, sodium β-glycerophosphate, Alizarin Red S Staining Solution, 4% paraformaldehyde solution, and 25% glutaraldehyde solution were obtained from solarbio (Beijing, China). α-MEM medium, penicillin/streptomycin (P/S, 1%), and fetal bovine serum (FBS) were purchased from gibico (Australia). Human BMP-2 ELISA Kit was obtained from Xinbosheng (Beijing, China). BCIP/NBT alkaline phosphatase (ALP) color development kit and CCK-8 kit were purchased from Biyuntian (Shanghai, China). ALP test kit was obtained from Jiancheng Biotechnology (Nanjing, China). Ultrapure water with a resistivity of 18.2 MΩ cm was used throughout.

### Instruments

Transmission electron microscopy (TEM) images were acquired on Tecnai T12 Cryo–electron microscope (FEI) operating with an acceleration voltage of 120 kV. Dynamic light scattering (DLS) measurements were performed on a Zetasizer Nano instrument (Malvern) with a 10-mW helium-neon laser and thermoelectric temperature controller. TMS was used as internal standard, and 1H nuclear magnetic resonance (NMR) spectra were recorded on a 500-MHz Bruker AV400 spectrometer. FTIR measurements were performed using an FTIR spectrometer (Nicolet 6700, Thermo). X-ray photoelectron spectroscopy measurements were performed using an x-ray photoelectron spectroscopy (ESCALAB 250Thermo). Fluorescence intensity imaging was performed using an Automatic multi-function imaging analysis system (OI600-MF-Touch). Cell adhesion on the surface of the material was observed by SEM (CarlZeiss SMT, Germany) imaging. Fluorescence intensity was measured with microplate reader Multiskan GO (Thermo). Fluorescently stained cells were imaged with an OLYMPUS upright fluorescence microscope (Japan). UV absorption measurement was used by NANODROP (Thermo).

### Cell Culture

Bone marrow mesenchymal stem cells (BMSCs) isolated from Sprague–Dawley (SD) rats were used to evaluate the *in vitro* cytocompatibility of the materials. The use of animals was performed in compliance with the ethical requirements of experimental animals. Bone marrow mesenchymal stem cells (BMSCs) was cultured on 25 cm^2^ tissue culture flasks and maintained by α-MEM, supplemented with 10% FBS and 1% P/S.

### Synthesis of the Amphiphilic Graft Polymer

The amphiphilic graft polymer was synthesized by conjugating HOOC-PEG-N_3_ and oleic acid to the side chain of PLL (Mw 30–70 kDa). Briefly, 90 mg of HOOC-PEG-N_3_, 6.4 µl of oleic acid, 12.8 mg of EDC, and 8.46 mg of sulfo-NHS were dissolved in 2 ml of DMF/NaHCO_3_ mixed solvent (volume ratio = 1:1) in sequence, and magnetically stirred for 1 h. Next, 7.5 g of PLL was added to the solution and continued to react for 24 h, followed by extensive dialysis against 30% ethanol solution using cellulose membrane (14 kDa) at room temperature for 48 h. Finally, OA-PLL-PEG-N_3_ (denoted as was OPP) was obtained by freeze-drying using a freeze dryer (FD-1A-50, Hangzhou Chuanyi). In this experiment, to explore the effect of buffer pH on the grafting efficiency, the volume ratio of the DMF:NaHCO_3_ mixed solution was adjusted to 3:1 (pH: 7.5–8.0), and the other experimental steps remained unchanged, to obtain OA-PLL-PEG- N_3_ IV (denoted as OPP Ⅳ).

### Characterization of the Amphiphilic Graft Polymer

To characterize the conjugation of OA to the PLL, the fluorescence spectroscopy was used to identify the presence of hydrophobic area in OPP. Briefly, different Nile Red solutions and 2 mg/ml OPP solutions were mixed and incubated at 4°C for 2 h. Then, the fluorescence of each solution was measured with a fluorescence spectrometer under the following conditions: an excitation wavelength of 500 and an emission wavelength of 800. To confirm the successful conjugation HOOC-PEG-N_3_, PLL the OPP, and PLL were mixed and compressed with potassium bromide, respectively, and then, they were detected by an FTIR spectrometer (Nicolet 6700, Thermo) at a wavelength of 400–4000 cm^−1^ to further determine the presence of PEG-N3 on OPP. Through a nuclear magnetic resonance spectrometer (AVANCE III, Brooke), at 500 MHz frequency, with TMS as internal standard and deuterated methanol as solvent, the NMR spectrum of OPP was obtained.

### Modification of the PCL Scaffold With Amphiphilic Graft Polymer

OPP was dissolved in 20% ethanol PBS solution at a final concentration of 2 mg/ml. The PCL scaffold was cut into a square with a side length of 1 cm firstly. Next, the PCL surface was rinsed with PBS and soaked in 1 ml of OPP solution, followed by incubation at 37°C for 3 h. Then, the PCL was taken out, rinsed with PBS three times, and placed in a vacuum oven at 37°C for 72 h.

### Synthesis of the Non-degradable BSA Nanocapsules

The non-degradable BSA nanocapsules, denoted as n(BSA)^ND^, were synthesized as previously reported (Supplementary Figure S2A). Briefly, 240 μl of BSA (6 mg/ml), 40 μl of acrylamide (AAm, 20% m/v), 12 μl of APm (20% m/v), and 1.7 μl of BIS (10% m/v) were thoroughly mixed in a 10 mM pH 7.00 PBS buffer. Free-radical polymerization was initiated by adding 11 μl of APS (10% m/v) and 3 μl of TEMED (10% m/v). The reaction was allowed to proceed for 2 h at 4°C and then was extensively dialyzed against 10 mM pH 7.0 PBS buffer using a cellulose membrane (MWCO 10 kDa) to remove unreacted monomers and initiators.

### Synthesis of the Degradable BSA Nanocapsules

The degradable BSA nanocapsules, denoted as n(BSA), were synthesized with a similar method to that of n(BSA)^ND^. Instead of using non-degradable cross-linkers, GDMA and Poly (DL–lactide)–b–poly (ethylene glycol)–b–Poly (DL–lactide) diacrylate (denoted as PLA-PEG-PLA diacrylate) were employed as the degradable cross-linker in the polymerization reaction. The rest of the steps are the same as n(BSA)^ND^.

### Synthesis of DBCO-Modified BSA Nanocapsules

The modification of n(BSA)^ND^ or n(BSA) was achieved by the reaction of the amine groups on the surface of the nanocapsules with DBCO-PEG-NHS. Briefly, BSA nanocapsules and DBCO-PEG-NHS were mixed thoroughly at a molar ratio of 1:25 (BSA to DBCO-PEG-NHS) and incubated at room temperature for 2 h. Then, the mixture was extensively dialyzed against 10 mM pH 7.0 PBS buffer using a cellulose membrane (MWCO 10 kDa) to remove any unreacted DBCO-PEG-NHS. The yielded DBCO-modified BSA nanocapsules were stored at −80°C for future use.

### Synthesis of the Fluorescence-Labeled BSA and Fluorescence-Labeled BSA Nanocapsules

For imaging purposes, BSA was fluorescently labeled with FITC (Ex 490 nm, Em 525 nm). Briefly, BSA and FITC are mixed at a molar ratio of 1:5 (BSA to FITC) and incubated at room temperature for 48 h under dark. Then, the mixture was extensively dialyzed against 10 mM pH 7.0 PBS buffer using a cellulose membrane (MWCO 10 kDa) to remove unreacted FITC and finally obtain the FITC-labeled BSA (denoted as BSA-FITC). Fluorescence-labeled BSA nanocapsules and fluorescence-labeled DBCO-modified BSA nanocapsules were synthesized according to the methods described above using BSA-FITC as the protein core.

### Synthesis of the DBCO-Modified Degradable BMP-2 Nanocapsules

The synthesis methods of degradable BMP-2 nanocapsules [denoted as n(BMP-2)] and DBCO-modified degradable BMP-2 nanocapsules [denoted as DBCO-n(BMP-2)] are the same as those of degradable n(BSA) and degradable DBCO-n(BSA) except BMP-2 was used as the protein core.

### Determination of the Protein Concentrations

The protein concentration of native protein and protein nanocapsules were determined by bicinchoninic acid (BCA) colorimetric protein assay according to the manufacturer’s protocol. Briefly, a tartrate buffer (pH 11.25) containing 25 mM BCA, 3.2 mM CuSO_4_, and protein/nanocapsule samples was incubated at 60°C for 30 min. After the reaction was cooled to room temperature, the absorbance reading at 562 nm was determined with a UV/Vis spectrometer. The measured protein was used as a standard.

### Characterizations of the Protein Nanocapsules

The particle size distribution of n(BSA)^ND^ and n(BMP-2) was obtained by measuring suspension using dynamic laser scattering (DLS, ZS90, Malvern, United Kingdom). The morphology of n(BSA)^ND^ and n(BMP-2) was evaluated by a transmission electron microscope (TEM, JZM-2100, Japan). In the TEM observation, the specimens were prepared by dropping a drop of 0.1% n(BSA)^ND^ and n(BMP-2) on a copper and observed at an acceleration voltage of 120 kV. The numbers of the DBCO groups in the DBCO-modified nanocapsules were determined by measuring the adsorption at 309 nm using an ultraviolet spectrophotometer (NAODROP Thermo) at a protein concentration of 0.5 mg/ml. The numbers of DBCO in DBCO-modified nanocapsules was calculated based on the Beer-Lambert law as:

$$Number\;DBCO\;Per\left(DBCO-\;nanocapsules\right)=\frac{A309}{\varepsilon 309\;\times Concentration\;of\;DBCO-\;nanocapsules}$$

Wherein A309 is the absorbance of DBCO-modified nanocapsules at 309 nm; ɛ309 represents the extinction coefficient of DBCO at 309 nm (ɛ309 = 12,000 M^−1^ cm^−1^).

### Preparation of PCL-OPP Modified With Protein Nanocapsules

To prepare PCL modified with protein nanocapsules, PCL-OPP was firstly soaked in BSA solution (30 mg/ml) and incubated at 37°C for 2 h to block non-specific adsorption. Next, the surface of PCL-OPP was rinsed with PBS, and then incubated with 1 ml of n(BSA)^ND^, DBCO-n(BSA)^ND^, DBCO-n(BSA), or DBCO-n(BMP-2) solution at a concentration of 2 mg/ml, respectively. After reacting overnight at 4°C, the surface was rinsed gently with PBS buffer, and placed in a 37°C vacuum oven (DZF-6021, Shanghai Yiheng) to allow vacuum drying for 72 h to obtain PCL modified with protein nanocapsules, which was denoted as PCL-n(BSA)^ND^, PCL-n(BSA), or PCL-n(BMP-2), respectively.

### Characterizations of PCL, PCL-OPP, and PCL-n(BSA)^ND^

To confirm that the OPP was successfully coated onto the PCL, x-ray photoelectron spectroscopy (59 ESCALAB 220i, Thermo Fisher Scientific, Inc., Waltham, MA, United States) was used to determine the chemical composition of PCL and PCL-OPP, and a scanning electron microscope (CarlZeiss SMT, Germany) was used to observe the morphology changes. Optical imaging was used to confirm the specific conjugation of fluorescence-labeled n(BSA)^ND^ to the PCL-OPP using an automatic multifunctional imaging analysis system (OI-600MF-Touch, Guangzhou Guangyi Biology).

### Water Contact Angle Assay

Water contact angle assay was used to test the hydrophilicity of the PCL, PCL-OPP, and PCL-n(BSA)^ND^ using the sessile drop method on a contact angle goniometer (DSA100, Dataphysics Instruments GmbH, Filderstadt, Germany). Briefly, the material was placed on the stage and raised so that the material to be tested will make contact with the water droplets of the injector. Then, the stage was lowered to take the water drop away. Finally, a picture of the water droplets was taken after a pause of 10 s. The five-point fitting method was used to measure the static water contact angle.

### Protein Adsorption Assay

The PCL and PCL-OPP were cut into 1-cm squares and respectively immersed in 1 ml (0.3 mg/ml) of BSA-FITC solution. After incubation at 37°C for 2 h, the surface of PCL and PCL-OPP was rinsed with 1 × PBS gently, and then the fully automatic multifunctional imaging analysis system (OI-600MF-Touch, Guangzhou Guangyi Biology) was carried out for fluorescence detection. The results were quantified by a Gel-Pro Analyzer.

### Protein Release Assays of the PCL-n(BSA) and PCL-n(BMP-2)

To quantify the BSA released from PCL-n(BSA), the square of PCL-n(BSA-FITC) was placed in 500 μl of 1 × PBS (pH 7.0) solution and incubated in a constant temperature water bath at 37°C. The solution was replaced with 500 μl of 1 × PBS (pH 7.0) solution every 24 h for 4 days. The fluorescence intensity of each collected sample was detected by a fluorescence microplate reader (Multiskan GO, Thermo). The release curve of BSA-FITC was calculated based on the time-dependent fluorescence intensity changes. For quantifying the release kinetics of BMP-2, 1 × 1 cm^2^ PCL-n(BMP-2) was placed in 400 μl of 1 × PBS (pH 7.0) solution. The 400 μl of PBS buffer was taken out at 1, 2, 3, 4, 5, 7, 10, 15, and 21 days for analysis using the BMP-2 Elisa kit according to the manufacturer’s protocol. Sample O.D. values at 450 nm were recorded by the microplate reader for calculating the release curve of BMP-2.

### Cytotoxicity Assays

BMSCs were extracted from newborn SD rats and then cultured with α-MEM (minimum essential medium eagle—alpha modification) with 10% FBS. Fourth-generation BMSCs were used to study the interaction between cells and nanocapsule shells. The BMSCs were allowed to adhere at a density of 5 × 10^3^ cells per well and incubated for 24 h. Then, 100 μl of n(BSA)^ND^ solution (20, 50, 150, 200, and 250 ng/ml), which were diluted with the serum-free medium in advance, were added. After the incubation for 24 h, 10 μl of cck8 was added to each well and the absorbance at 450 nm was measured by a multifunctional microplate reader (EnSpire PerkinElmer). Taking the cell viability of the absorbance value at the blank unmedicated hole as 100%, the relative cell viability of the transformed experimental group.

### Osteogenic Differentiation Assay of BMSCs

Second-generation BMSCs were used to study the interaction between cells and n(BMP-2). The BMSCs were seeded to adhere at a density of 2.5 × 10^4^ cells per well and cultured in a carbon dioxide cell incubator (311 Thermo) for 24 h to allow the cells to attach. On the basis of the conventional culture medium, the osteogenic induction medium containing 0.1 μM dexamethasone, 0.05 mg/ml ascorbic acid, and 10 mM sodium glycerophosphate aqueous solution was prepared for subsequent culture. The concentration of n(BSA) and n(BMP-2) in each well of the control or experimental group was maintained at 500 ng/ml per well, and nothing was added to the blank control group. The liquid was changed every 3 days. Then, the cells were stained with the BCIP/NBT ALP staining kit on the third day and stained with Alizarin Red on the 21st day subsequently. All the staining results were observed under an optical microscope and quantitatively analyzed by Image-Pro software.

### Adhesion and Proliferation Assay of BMSCs on PCL, PCL-OPP, and PCL-n(BSA)^ND^

Fourth-generation BMSCs were used to study the cell adhesion and proliferation on PCL, PCL-OPP, and PCL-n(BSA)^ND^. The PCL and PCL and PCL-OPP were disinfected in 75% ethanol for 2 h and rinsed three times with sterile PBS. PCL-n(BSA)^ND^ was obtained from the above sterile PCL-OPP and DBCO-n(BSA)^ND^ according to the method described above and equilibrated it in a culture medium for 2 h. The BMSCs were allowed to adhere to the material’s films for 4 h at a density of 2.5 × 10^5^ cells per well. After 8 h, the films were washed three times with PBS to remove any loosely attached cells, 0.5 ml of culture medium containing 10% CCK-8 was added, and the optical densities at 450 nm were measured. The CCK-8 assay was also carried out at 2 and 4 days. On the third day, the medium was aspirated from each well and fixed with 2.5% glutaraldehyde for 15 min. The cell adhesion on the surface of the material was observed by SEM (CarlZeiss SMT, Germany) imaging, and the cell adhesion area was quantified using ImageJ software. The adhesive of BMSCs to the PCL, PCL-OPP, and PCL-n(BSA)^ND^ was observed at different time intervals using fluorescence imaging (OLYMPUS, Japan). Cells were stained by live and dead, and the number of live cells was stained to determine the number of adherent cells.

### Osteogenic Differentiation Assay of BMSCs Cultured on PCL, PCL-OPP, PCL-n(BSA), and PCL-n(BMP-2)

BMSCs were cultured on PCL, PCL-OPP, PCL-n(BSA) and PCL-n(BMP-2) with osteogenesis-inducing supplements for 21 days. Firstly, the cells were lysed by 0.2% Triton X-100 v/v for 30 min and then pipette all the lysate was pipetted. Cell homogenate to be tested was added into each well of the 96-well plate, 30 μl of water was added to the blank group, and 30 μl of phenol standard solution (0.02 mg/ml) was prepared for the standard group. After stopping the reaction, the microplate reader (Multiskan GO Thermo) detected the value of OD520. In addition, part of the homogenized cell homogenate was used to determine the total cell protein concentration by the BCA method, and the ALP activity was calculated according to the following formula (King’s unit/gprot):

$$AKP\;viability\;in\;cultured\;cell\left(King's\;unit/gprot\right)=\frac{OD1-OD2}{OD3-OD1}\times Phenol\;\mathrm{standard}\;concentration÷Total\;portein\;concentration$$

Wherein OD1 represents the absorbance value of the measuring hole, OD2 represents the absorbance value of the blank hole, and OD1 represents the absorbance value of the standard.

### Alizarin Red Staining Assay of BMSCs Cultured on PCL, PCL-OPP, PCL-n(BSA), and PCL-n(BMP-2)

The mineralization of BMSCs was assessed at day 21 using an Alizarin Red Staining (ARS) assay. The BMSCs were rinsed three times with PBS and fixed with 4% of paraformaldehyde solution for 15 min, subsequently stained with alizarin red for 30 min, and then rinsed with ultrapure water three times. The calcium deposition was observed using Biological Scanning Electron Microscope (SU8010 Thermo).

### Statistical Analysis

All results are presented as the mean ± standard error of the mean (s.e.m.) as indicated. Two-way ANOVA was used for multiple comparisons. All statistical analyses were conducted with Prism Software (Prism 8.0.1).

## Results and Discussions

### Synthesis and Characterization of the Amphiphilic Graft Polymer

Biocompatibility and robust mechanical strength both are the fundamental concerns for nano fabric scaffolds used in tissue engineering. For scaffolds made of hydrophobic polymers, such as commonly used polyesters and polyethylene derivatives, surface biofunctionalization is an efficient method to enhance biocompatibility without compromising the mechanical property. In this work, we synthesized an amphiphilic graft polymer based on fully biodegradable PLL. The graft PLL polymer was designed to contain a 50% molar ratio of oleic acid as the hydrophobic moieties for interaction with nanofibers. The remaining 50% of side chains are left for conjugation with poly(ethylene glycol) terminally modified with azide group (PEG-N_3_) (Supplementary Figure S1A). For stable coating of the hydrophobic scaffold, PLL with 240 repeat units was chosen (Average Mw: 50 kDa of the PLL hydrobromide). The polymer main chain could thus provide theoretically 120 alkyl chains for multiple van der Waals interactions and 120 PEG-N_3_ for multivalent biofunctionalization, respectively.

**FIGURE 1:**
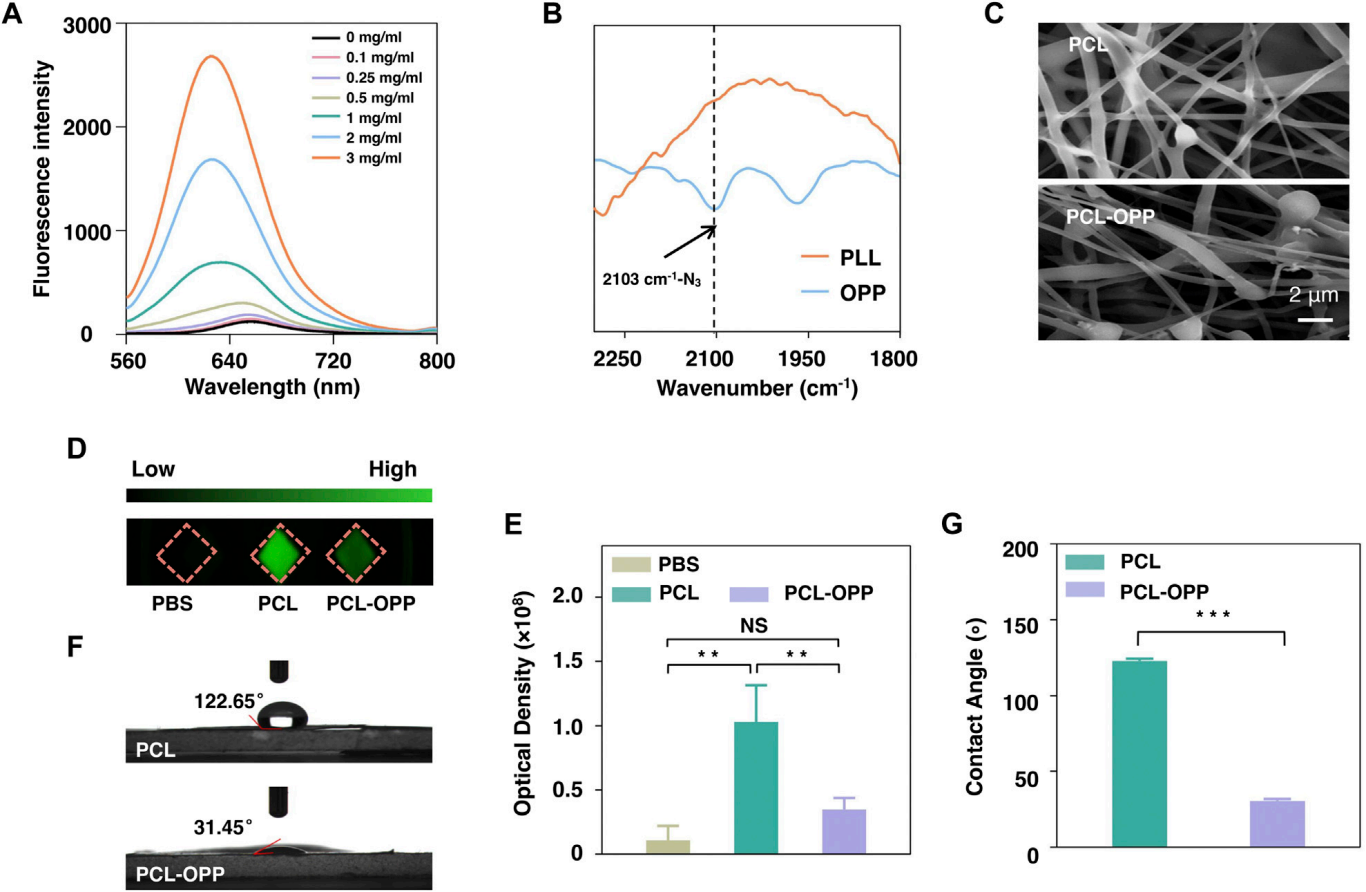
Characterization of PCL and PCL-OPP. **(A)** Fluorescence spectra of Nile Red after incubation with different concentrations of OA-PLL-PEG-N_3_(OPP). **(B)** FT-IR spectra of PLL and OA-PLL-PEG-N_3_(OPP). **(C)** SEM image of PCL and PCL-OPP. **(D)** FITC-labeled BSA protein adsorption. (E) Quantitative fluorescence density analysis of the BSA-FITC protein adsorption using Image-Pro software. **(F, G)** The water contact angle of PCL and PCL-OPP. (*n* = 3). *p* value: *p < 0.05, **p < 0.01, ***p < 0.001 and ****p < 0.0001. ns means not significant.

By optimizing the coupling reaction mediated by 1-ethyl-3-carbodiimide, two batches of graft copolymer OA-PLL-PEG-N_3_ Ⅲ and OA-PLL-PEG-N_3_ Ⅳ were obtained. The products were characterized by ^1^H-NMR, showing characteristic chemical shifts of CH_2_ groups from PEG, PLL, and the double bond of oleic acid (OA) (Supplementary Figures S1B, C). For the product OA-PLL-PEG-N_3_ Ⅳ, the integral of these corresponding chemical shifts corresponded to 36% of OA and 20% of PEG conjugation on the PLL side chains. Therefore, OA-PLL-PEG-N_3_ Ⅳ with the highest proportion of oleic acid was selected for the following experiments, denoted as OPP. Fluorescence emission spectroscopy shows enhanced solubility of hydrophobic Nile Red dye by mixing with the grafted polymers, indicating the formation of amphiphilic polymer micelles by successfully coupling oleic acids to PLL-PEG (Figure 1A). Fourier transform infrared spectroscopy (FT-IR) of the graft polymer showed a new absorption at 2,103 cm^−1^ for the asymmetric stretch vibration of the azide group (Figure 1B). X-ray photon spectroscopy further proves the presence of azide groups in the complex (Supplementary Figure S1D), which were absent for PLL polymer before the coupling reaction. The results therefore unambitiously confirmed the successful synthesis of OA-PLL-PEG-N_3_, where the azide group could be used for click reaction for further bioconjugations.

### Preparation and Characterization of Surface-Modified PCL Scaffold

Next, we used the obtained amphiphilic graft polymer to coat the hydrophobic scaffold *via* dip-coating. For this purpose, PCL nanofiber membrane was first to cut into pieces of 1 × 1 cm^2^ for dipping into 2 mg/ml OA-PLL-PEG-N_3_ (hereafter denoted as OPP for short) PBS solution. Scanning electron microscopy (SEM) results showed that the fiber surface of the PCL-OPP had become rough with particles attached (Figure 1C). BSA-FITC was further used to study the protein adsorption capacity of PCL before and after modification. Figure 1D shows the fluorescence images of the PCL and PCL-OPP after incubation with BSA-FITC at 37°C for 2 h. The green fluorescence on PCL-OPP is weaker than that of PCL (Figure 1D), indicating a lower protein adsorption capacity of PCL-OPP. Quantitative analysis shows that the non-specific protein adsorption capacity of the PCL-OPP scaffold is 3-fold lower than that of the pure PCL scaffold (Figure 1E). This result indicates successful functionalization of OPP on PCL nanofiber membranes, which introduces a PEG layer on the PCL surface to inhibit non-specific protein adsorption. In agreement with this result, a significant decrease of the water contact angle was determined on PCL-OPP (31.45°) versus PCL of 122.65° (Figure 1F, G), corroborating the fact that the coating of OPP renders the PCL surface highly hydrophilic.

### Synthesis and Characterizations of BSA Nanocapsules

**FIGURE 2:**
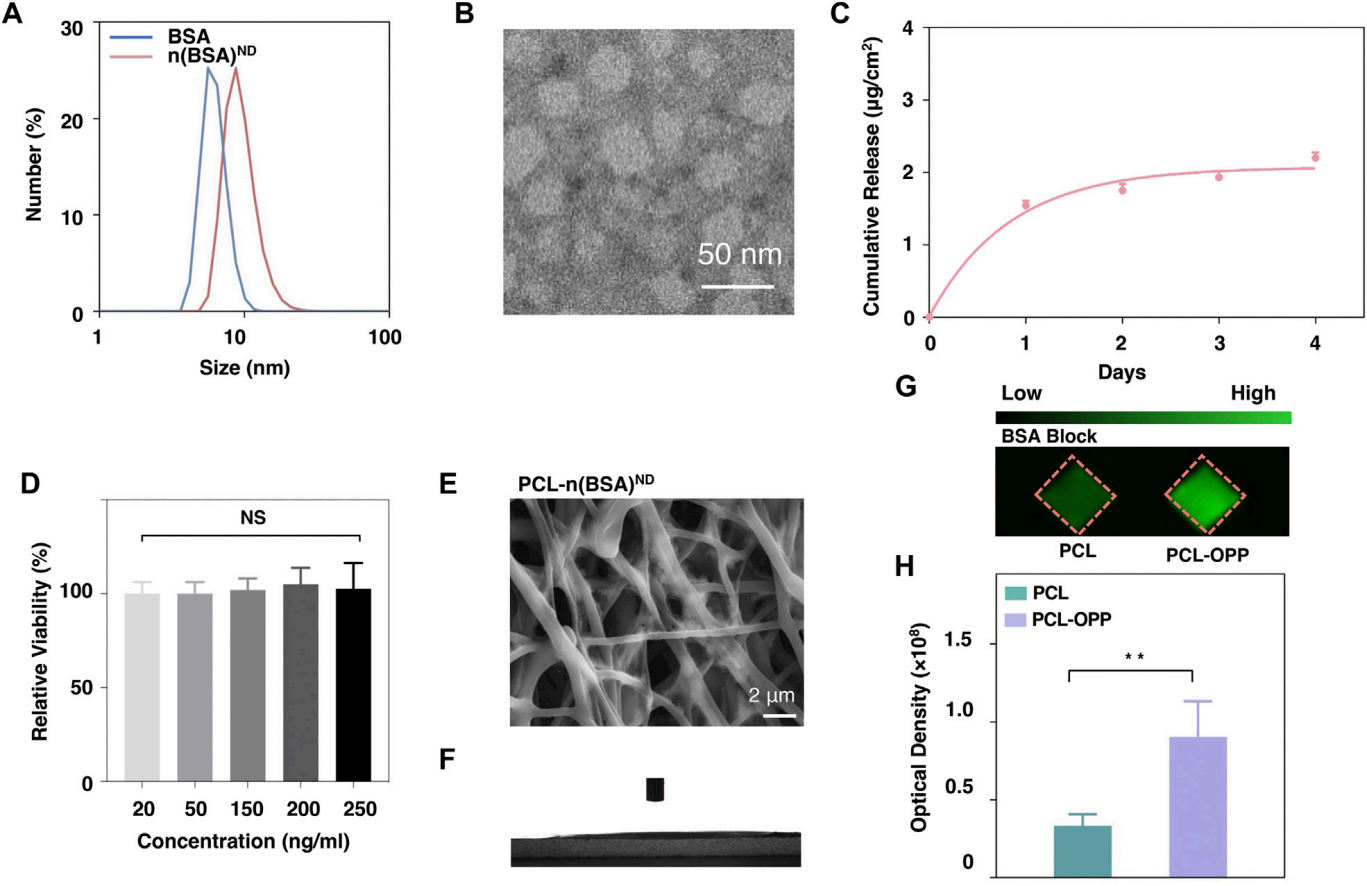
Characterization of n(BSA)^ND^, DBCO-n(BSA), and PCL-n(BSA)^ND^. **(A)** The size of BSA and n(BSA)^ND^. **(B)** Representative TEM image of negatively stained n(BSA)^ND^. **(C)** The release rate of the BSA cargo from DBCO-degradable n(BSA). **(D)** Cell viability on different concentrations of n(BSA)^ND^. **(E)** SEM image and **(F)** Water contact angle of PCL-n(BSA)^ND^. **(G)** Imaging of the PCL and PCL-OPP after incubation with DBCO-n(BSA-FITC)^ND^. **(H)** Quantification of the fluorescence density analyzed using Image-Pro software (*n* = 3, NS means no significant difference, *p < 0.05, **p < 0.01, ***p < 0.001).

To investigate whether the as-obtained PCL-OPP could be further functionalized *via* the azide group on the PEG side chain and demonstrate the synthesis of the nanocapsules with sustained-release capability, we prepared dibenzocyclooctyne (DBCO)-modified protein nanocapsules using BSA as the model protein nanocapsules and explored their coupling efficiencies on PCL-OPP. The synthesis of the model protein nanocapsules [denoted as n(BSA)] can be achieved by *in situ* polymerization at 4°C (Supplementary Figures S2A,B). DLS results showed that the hydrodynamic radius of BSA was 6.242 nm (Figure 2A), while the hydrodynamic radius of n(BSA)^ND^ reached 9.544 nm (Figure 2A), indicating that the AAm, APm monomers, and GDMA cross-linkers incubated with BSA had formed a thin layer of polymer network around the protein through free radical polymerization, leading to the formation of n(BSA). This was consistent with the TEM image showing that n(BSA)^ND^ had a spherical morphology with an average diameter of about 20 nm (Figure 2B). These results collectively suggested the successful synthesis of n(BSA)^ND^.

### Surface Modification of PCL-OPP With BSA Nanocapsules

Next, in order to explore the immobilization of nanocapsules on the surface of PCL-OPP material, DBCO-PEG-NHS ester was used to modify the primary amino groups (-NH_2_) on the surface of n(BSA) to form DBCO-n(BSA)^ND^. From the full-band scanning absorption peak of the UV spectrophotometer, it can be seen that there are obvious absorption peaks at 309 nm (Supplementary Figure S2C), indicating that DBCO was successfully grafted to the n(BSA)^ND^ surface. As-obtained DBCO-n(BSA)^ND^ can be specifically immobilized on the surface of PCL-OPP by reacting with the azide group on PCL-OPP (“Click Chemistry” reaction), which is denoted as PCL-n(BSA)^ND^. Figure 2E shows the SEM image of the PCL-n(BSA)^ND^. Compared with the surface of PCL-OPP, the surface of PCL-n(BSA)^ND^ has a relatively rough fiber structure. Moreover, the water contact angle of PCL-n(BSA)^ND^ is significantly lower than that of PCL-OPP (Figure 2F), so that the angle cannot be displayed. To further confirm that the DBCO-n(BSA)^ND^ was covalently conjugated rather than absorbed to the scaffold, the PCL and PCL-OPP were pre-incubated with BSA for block and further incubated with DBCO-n(BSA)^ND^, respectively. Figures 2G,H shows that the amount of specific immobilized protein on PCL-OPP is about twice that of PCL. Supplementary Figure S4 also proves that PCL-OPP performs specific immobilization rather than specific adsorption on DBCO-n(BSA)^ND^, and the protein immobilization capacity of the DBCO-n(BSA-FITC)^ND^ is about twice the non-specific adsorption capacity of the n(BSA-FITC)^ND^, suggesting that DBCO-n(BSA) efficiently and specifically immobilized on the PCL-OPP.

Under the psychological environment, the ester bonds in the cross-linkers in nanocapsules were gradually cleaved, leading to the dissociation of the polymer shells and the release of the protein cargo. The polymer shell composition can be readily altered to finely tune the degradation kinetics. We monitored the BSA concentration in PBS buffer (pH 7.0) over days from a PCL-n(BSA-FITC) nanofiber membrane leading to the controllable release. Through the BSA release curve, one can see that n(BSA) had sustainable release capability (Figure 2C) to avoid the sudden release of protein. Through the cytotoxicity test of different concentrations (20–250 ng/ml) of n(BSA)^ND^, the nanocapsule shell has no obvious cytotoxicity (Figure 2D), which is beneficial for the *in vivo* application of nanocapsules.

### BMSCs Adhesion and Proliferation on PCLn(BSA)ND Scaffold

**FIGURE 3:**
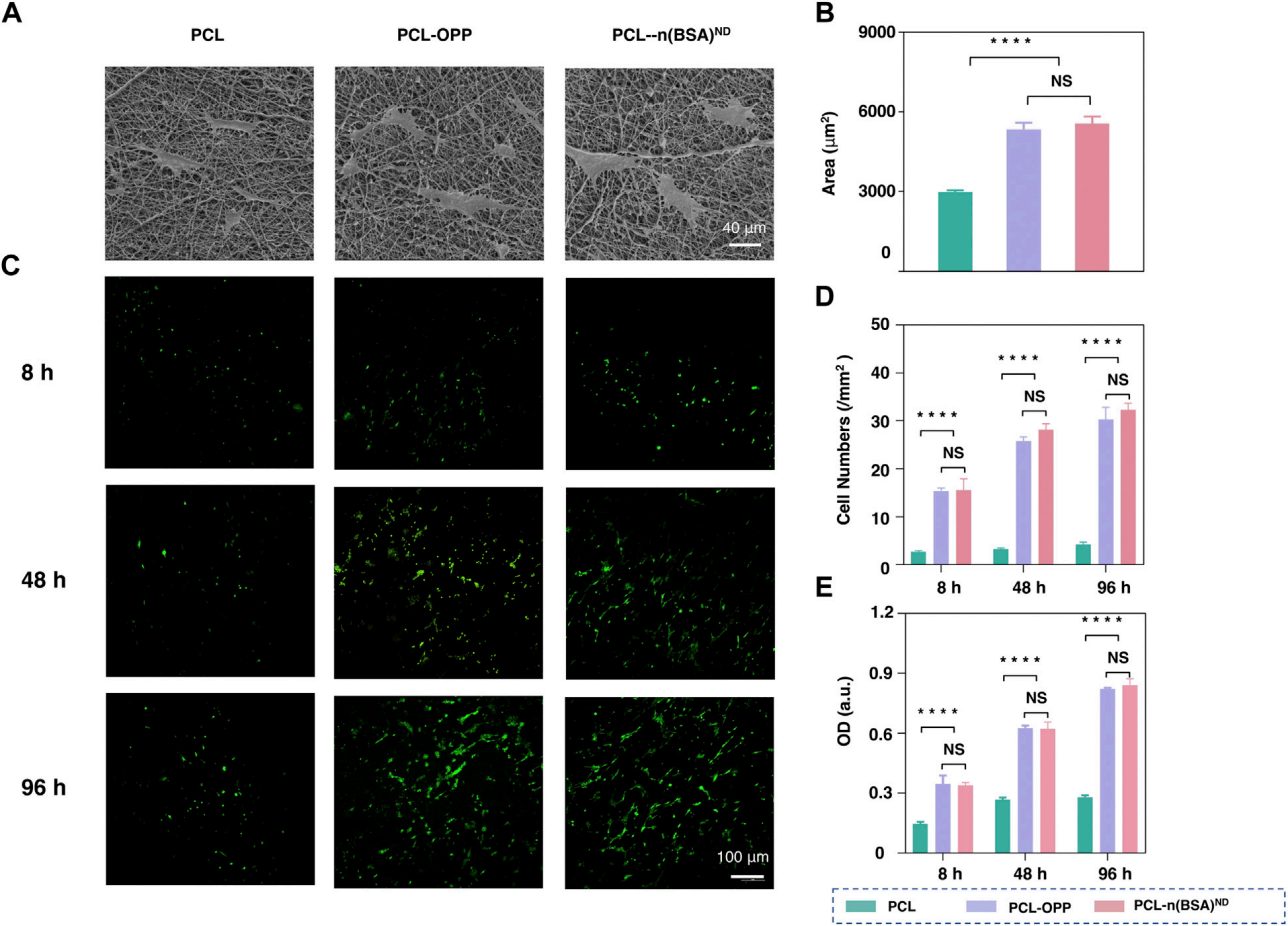
The adhesion and cell proliferation of BMSCs on PCL-n(BSA)^ND^. **(A)** SEM images of BMSCs adhered to the surfaces of PCL, PCL-OPP, and PCL-n(BSA)^ND^ films on the third day. **(B)** Quantitative analysis of cell area adhered to the surfaces of PCL, PCL-OPP, and PCL-n(BSA)^ND^ using ImageJ software. **(C)** The fluorescence images of BMSCs adhered to the surfaces of PCL, PCL-OPP, and PCL-n(BSA)^ND^ films at different time points. **(D)** Quantitative analysis of the cell numbers adhered to the surfaces of PCL, PCL-OPP, and PCL-n(BSA)^ND^ using ImageJ software. **(E)** Profiles of BMSC proliferation obtained by CCK-8 assay (*n* = 3, NS means no significant difference, ****p < 0.0001).

To test the biocompatibility of PCL-n(BSA)^ND^ of cell culture, BMSCs were chosen to study the effect of surface-modified PCL membrane on cell behaviors such as cell adhesion and proliferation. Scanning electron microscope images were used to observe the adhesion areas of BMSCs on the PCL, PCL-OPP, and PCL-n(BSA)^ND^ film surfaces on the third day (Figure 3A). The area of cell adhesion on PCL-OPP and PCL-n(BSA)^ND^ materials is much larger than that of the PCL group, which is about twice that of the unmodified PCL nanofiber membrane (Figure 3B).

Fluorescence microscopy images were used to observe the adhesion and growth of BMSCs on the PCL, PCL-OPP, and PCL-n(BSA)^ND^ membranes. As shown in Figure 3C, after 8 h of adhesion, only a few BMSCs adhered to the PCL membrane, and the number of cells that adhered to the PCL-OPP and PCL-n(BSA)^ND^ membranes was several times higher than that of the PCL group. As the culture time increases, not only does the number of cells on the surface of the PCL-OPP and PCL-n(BSA)^ND^ films increase but also the cells show good morphology for a spreading growth. In contrast, the number of cells on the surface of the PCL membrane only increased slightly compared to the 8 h. Since the number of cells adhering to the surface of the PCL membrane was too small in the first 8 h, even though the culture time was as long as 4 days, the number of cells on the PCL film surface was still several times lower than the PCL-OPP and PCL-n(BSA)^ND^ groups. Quantitative data on the number of adherent cells also verified this trend (Figure 3D).

The Cell Counting Kit-8 (CCK-8) test was used to further test the adhesion and proliferation of BMSCs on PCL, PCL-OPP, and PCL-n(BSA)^ND^ membranes. As shown in Figure 3C, the optical density (OD) values of the PCL-OPP and PCL-n(BSA)^ND^ groups in each period were significantly higher than those of the PCL group. No significant difference was found between the PCL-OPP and PCL-n(BSA)^ND^ groups. The CCK-8 assays were further used to test the adhesion and proliferation of BMSCs on the PCL, PCL-OPP, and PCL-n(BSA)^ND^ films. As seen in Figure 3E, the OD values of the PCL-OPP and PCL-n(BSA)^ND^ groups in each period were significantly higher than that of the pristine PCL group, and there was no significant difference between the PCL-OPP and PCL-n(BSA)^ND^ groups in all the above data. All results proved that the modification of PCL surface with OPP had improved the adhesion and proliferation ability of BMSCs on the material, which was consistent with the previous conclusion that PCL-OPP was significantly more hydrophilic than PCL. The results showed that immobilized nanocapsules did not affect the adhesion and proliferation of BMSCs on PCL-OPP, which pave the way for further application of PCL-OPP material loaded with functional growth factor nanocapsules.

### Synthesis and Characterizations of BMP-2 Nanocapsules

**FIGURE 4:**
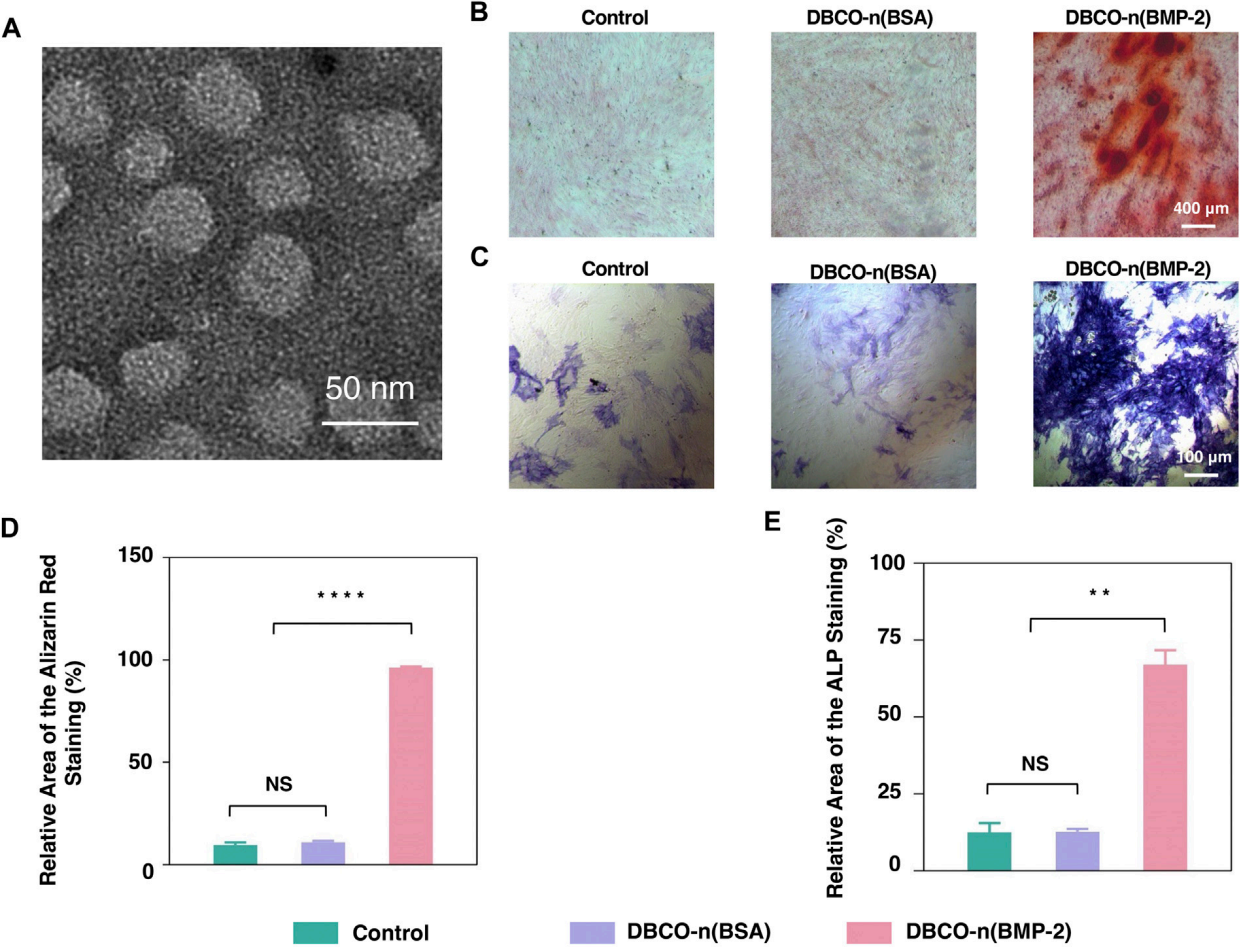
Osteogenic differentiation of BMSCs by n(BMP-2). **(A)** Representative TEM image of negatively stained degradable n(BMP-2). **(B)** Alizarin Red Staining of induced BMSCs for 10 days. **(C)** The ALP staining of induced BMSCs. **(D)** Quantitative analysis of Alizarin Red Staining images using ImageJ software. **(E)** Quantitative analysis of ALP staining images was analyzed using ImageJ software (*n* = 3, NS means no significant difference, **p < 0.01, ****p < 0.0001).

Inspired by the successful preparation of the PCL-n(BSA) nanofiber scaffold, n(BMP-2) was synthesized with a similar method to n(BSA). A purified sample was analyzed by SDS-PAGE, in which the bands of product are stacked at the junction of the concentrated gel and the separating gel (Supplementary Figure S3A). By the formation of a polymer shell on n(BMP-2), the molecular weight increased greatly, and it could not migrate in the gel as easily as BMP-2. To the end, no obvious BMP-2 band was found after purification of n(BMP-2) (Supplementary Figure S3A). DLS results showed that the diameter of n(BMP-2) reached 19.28 nm (Supplementary Figure S3B), which was consistent with the result provided by the TEM image, showing a spherical shape with an average diameter of about 20 nm (Figure 4A). Together, the above results confirmed the successful synthesis of n(BMP-2).

### Effect of BMP-2 Nanocapsules on BMSC Osteogenic Differentiation

ALP is a specific marker in the early stage of osteogenic differentiation. Meanwhile, calcium deposition is one of the important signs of late osteogenic differentiation, which can be specifically read out by ARS. To quantify the effect of BMP-2 nanocapsules on osteogenic differentiation, ALP and ARS staining were carried out to BMSCs cultivated with osteogenic induction medium alone, with n(BSA) or n(BMP-2) in induction medium for 21 days, respectively. Figures 4B,C showed ALP staining with blue-purple and ARS staining of calcium deposits in red. By quantifying the staining area of ALP and ARS (Figures 4D,E), it can be seen that although the BMSC culture always contains the induction medium, the percentage of ALP-stained area for the control group and the n(BSA) added group were about 12%, and the proportion of the ARS-stained area was only about 10%. In contrast, the ALP staining area of the experimental group added (BMP-2) accounted for 67.02%, and the ARS staining area accounted for 96.31%, which were both significantly higher than the n(BSA) group and the control group. These results collectively suggested that n(BMP-2) had excellent differentiation osteogenic differentiation ability.

**FIGURE 5:**
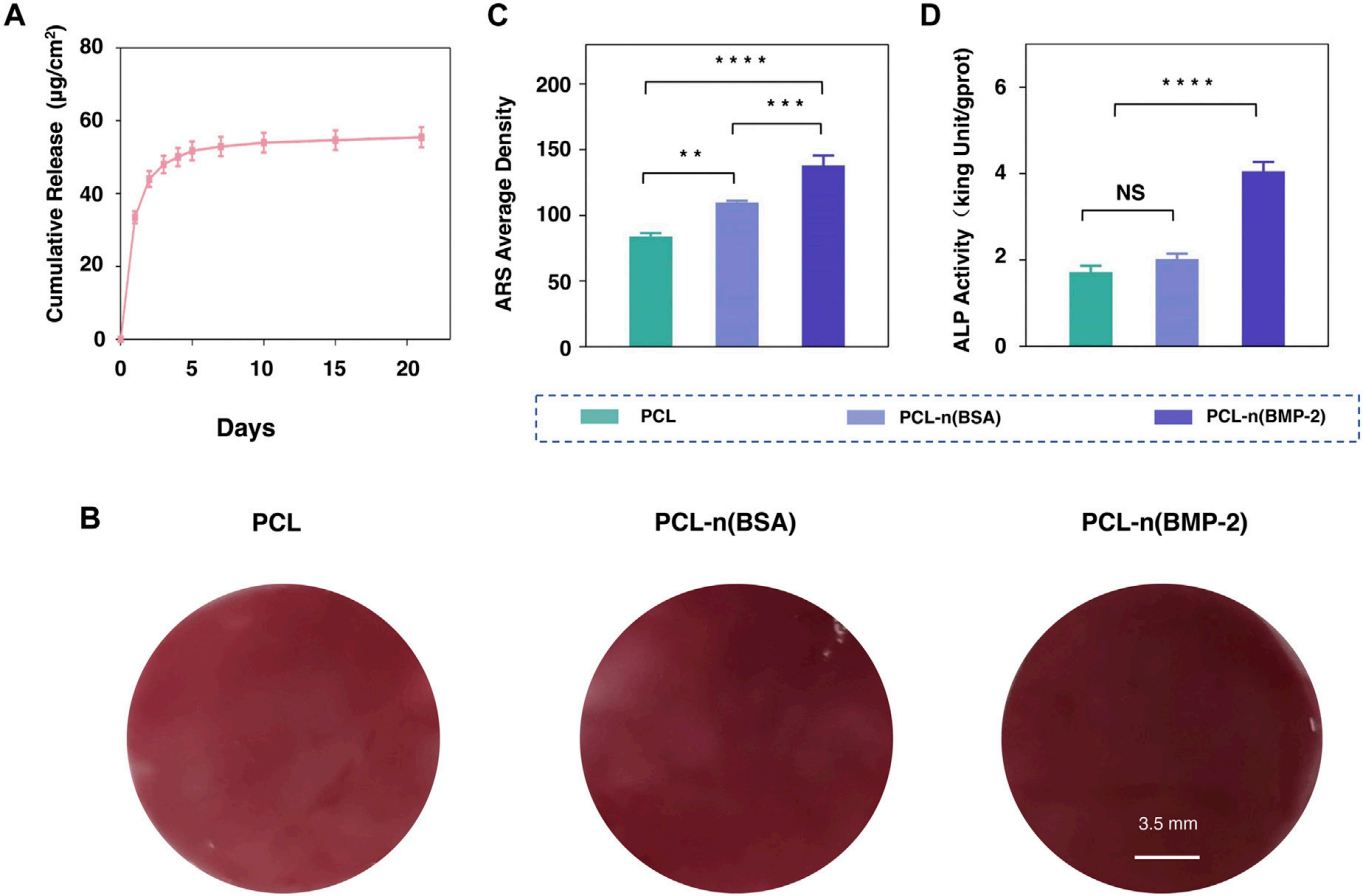
Osteogenic differentiation of BMSCs on PCL-n(BMP-2). **(A)** Release rate of the BMP-2 cargo from PCL-n(BMP-2). **(B)** Alizarin red staining assay of cultured BMSCs on PCL, PCL-n(BSA/BMP-2) at day 21. **(C)** The average density of Alizarin red staining was analyzed by ImageJ software for the quantification. **(D)** ALP activity of cultured BMSCs on PCL, PCL-n(BSA/BMP-2) osteogenic differentiation at day 3 (*n* = 3, NS means no significant difference, **p < 0.01, ***p < 0.001, ****p < 0.0001).

To obtain the sustained release of BMP-2 on biocompatible PCL scaffold, DBCO-n(BMP-2) was immobilized to PCL-OPP according to the abovementioned method and denoted as PCL-n (BMP-2). The effect of BMSC osteogenic differentiation on PCL-n(BMP-2) was evaluated by the above-established ALP and ARS staining. The release kinetics of BMP-2 from PCL-n(BMP-2) incubated in PBS at 37°C were monitored using a BMP-2 ELISA kit. According to this assay, the release of BMP-2 was increased gradually within 10 days. The release amount began to be steadily slow down after the first 10 days and continued until the 21st day as the end of the experiments (Figure 5A). The long-term controlled release of BMP-2 showed a cumulative release amount of 53.6 μg/cm^2^, which was sufficient to induce the osteogenic differentiation of BMSCs.

For evaluating ossification at the early differentiation stage, the ALP activity was detected on the third day after culturing BMSCs with PCL, PCL-n(BSA), and PCL-n(BMP-2). ARS assay was used to evaluate the synthesis of extracellular mineralized substances on day 21 for the late stage of differentiation. As shown in Figure 5B, although the material itself has a certain thickness, the calcium nodules can be detected through the microscope, the results showed that intensity of redness of ARS on PCL-n(BMP-2) was the strongest, followed by a sequence of PCL-n(BSA), and 1.8-fold higher than PCL (Figure 5C). Interestingly, the ALP activity results on day 3 showed the highest ALP activity of the PCL-n(BMP-2) group, which was twice that of the PCL and PCL-n(BSA) group (Figure 5D). These results are in a good correlation of the release curve of BMP-2, showing a stronger differentiation effect of BMP-2 in the early stage supported by the fast BMP-2 release. In line with the continuous release until day 21, the highest ARS at day 21 of the PCL-n(BMP-2) group highlights the importance of the long-time release of BMP-2 for the sustained ossification. In general, it furthermore illustrates the necessity of specific immobilization of functional signaling nanocapsules on PCL-OPP scaffold for applications in tissue engineering.

## Conclusion

In summary, we have demonstrated a general approach to surface modification of PCL scaffold with improved biocompatibility and controlled growth factor release for enhanced stem cell differentiation. Compared with PCL, the hydrophilicity of PCL-OPP has been significantly improved, thus providing improved biocompatibility, as well as better cell adhesion, and growth. In addition, through specific immobilization of BMP-2 nanocapsules onto the PCL-OPP, sustained release of BMP-2 enhanced osteogenic differentiation of BMSC. Moreover, as a general method, our strategy could be extended for bioactive functionalization of other hydrophobic scaffold materials with other therapeutic proteins in a variety of clinical applications.

## Data Availability

The original contributions presented in the study are included in the article/Supplementary Material. Further inquiries can be directed to the corresponding authors.
