# Supplementary material for: Surface Modification of Polycaprolactone Scaffold With Improved Biocompatibility and Controlled Growth Factor Release for Enhanced Stem Cell Differentiation
Source: Front Bioeng Biotechnol. 2022 Jan 7;9:802311. doi: 10.3389/fbioe.2021.802311 (PMC8782149; doi:10.3389/fbioe.2021.802311)
Supplement: Supplementary file 1 [file DataSheet1.DOCX]

Supplementary Material

## Supplementary Figure

##
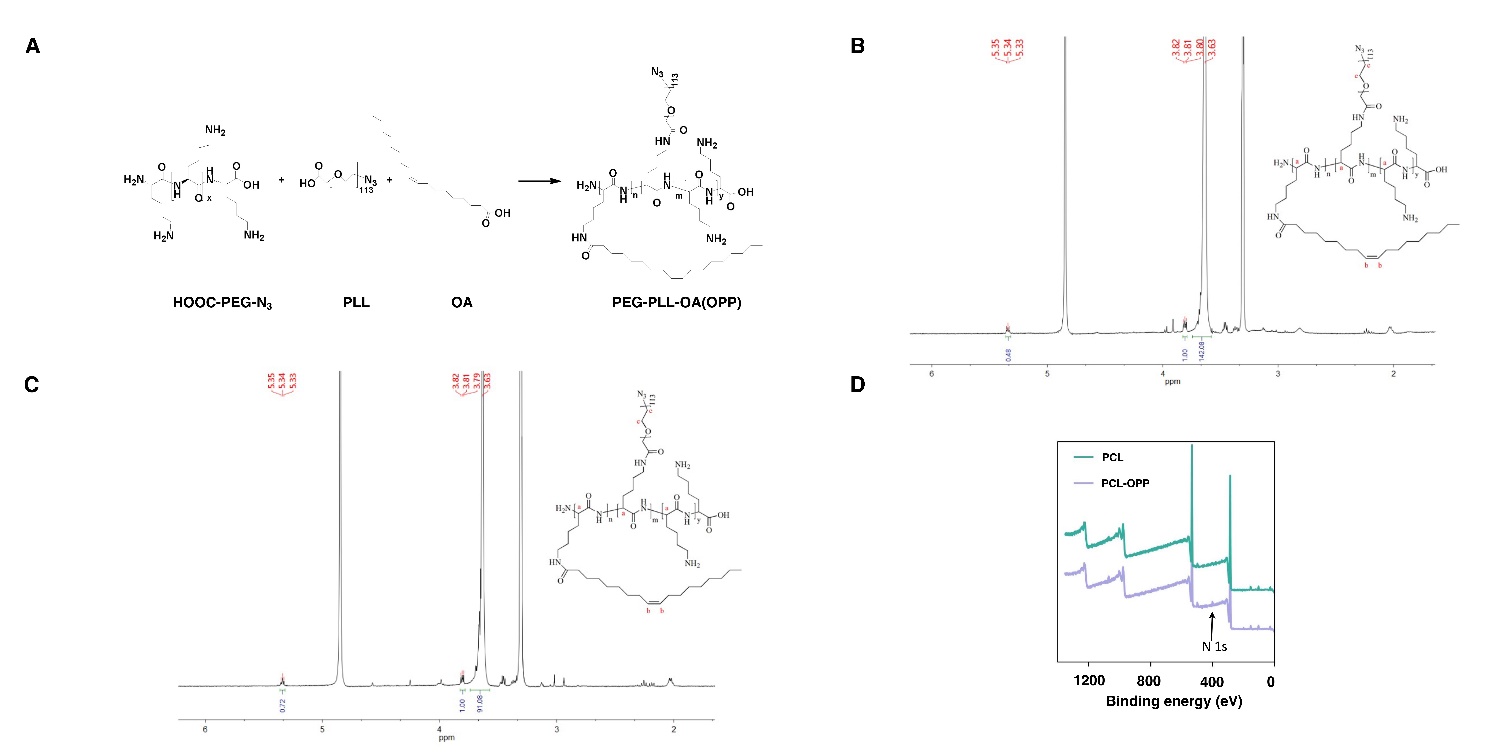


## Supplementary Figure 1. (A) Synthetic formula of amphiphilic graft polymer. (B,C) ^1^H NMR spectrum of OA-PLL-PEG-N3-Ⅲ (B) and OA-PLL-PEG-N3-Ⅳ(C). (D) XPS of PCL and PCL-OPP.


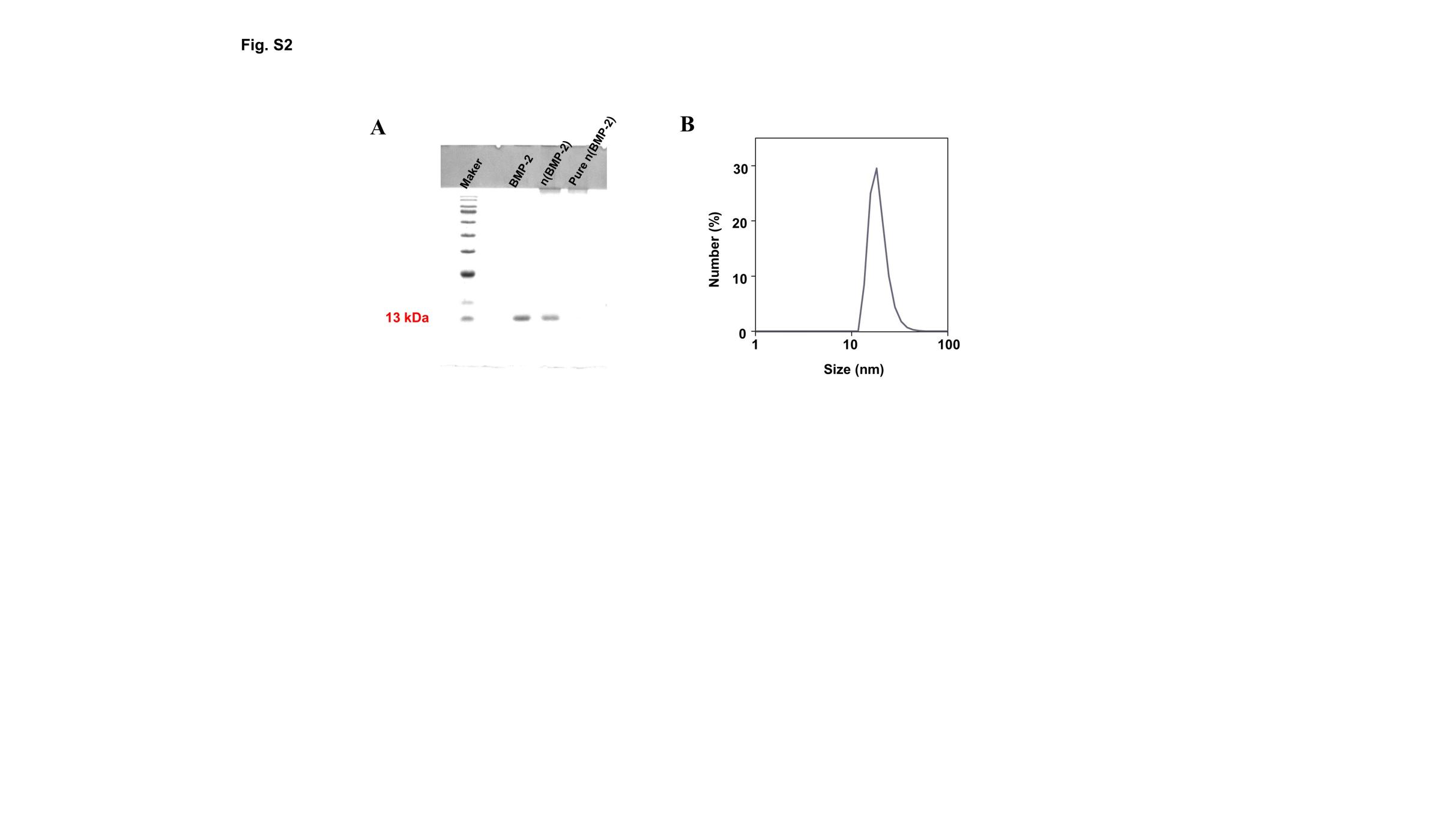


**Supplementary Figure 2.** (A) SDS-polyacrylmide gel electrophoresis of n(BMP-2). (B) The size distribution of n(BMP-2).


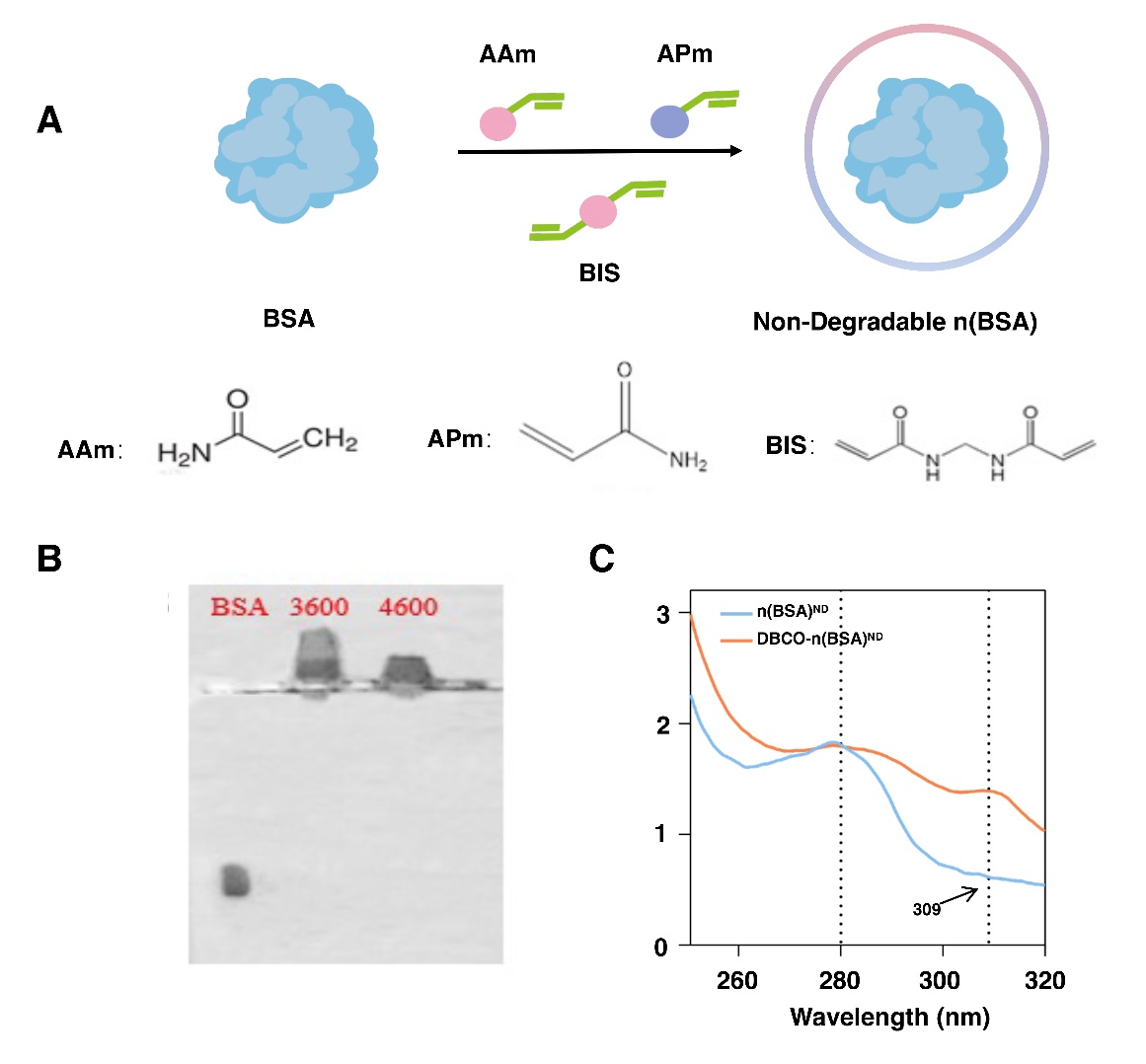


**Supplementary Figure 3.** (A) Schematic diagram of the synthesis of non-degradable BSA nanocapsules . (B) Agarose gel electrophoresis of n(BSA-FITC). (C) UV-VIS spectrum of n(BSA) and DBCO-n(BSA).


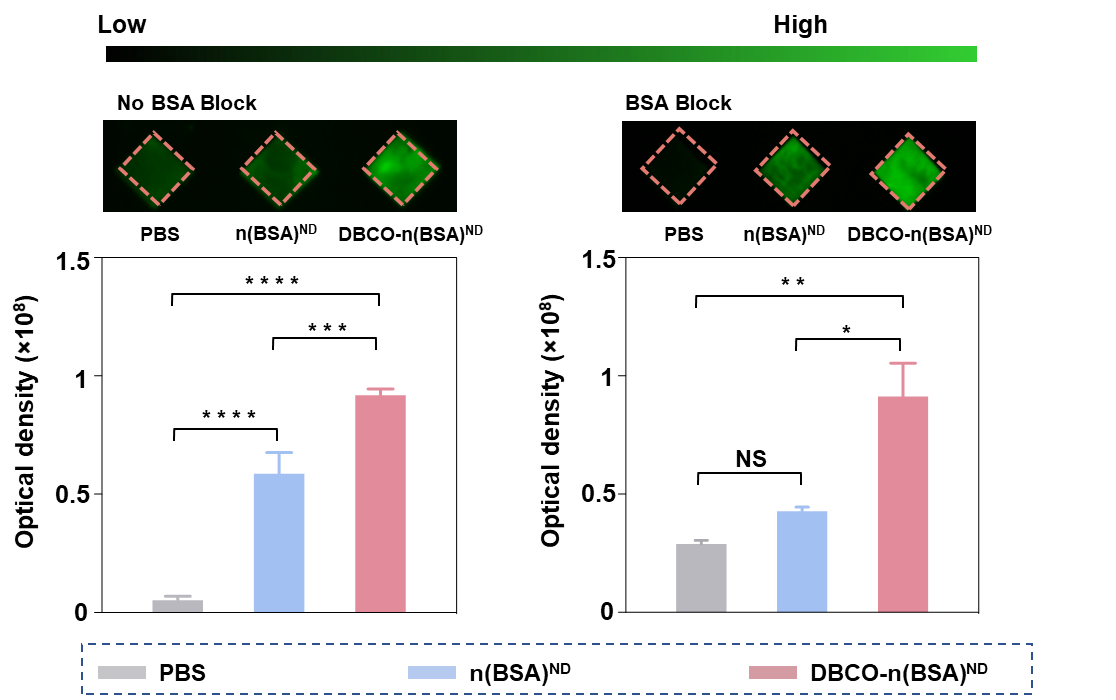


**Supplementary Figure 4.** The left panel is the specific loading capacity of DBCO-n (BSA-FITC) and n (BSA-FITC) to PCL-OPP without BSA Block.The right panel is the specific loading capacity DBCO-n (BSA-FITC) and n (BSA-FITC) to PCL-OPP with BSA block.
